# Supplementary material for: A Comparative Study on the Neuroprotective Effect of Geopung-Chunghyuldan on In Vitro Oxygen–Glucose Deprivation and In Vivo Permanent Middle Cerebral Artery Occlusion Models
Source: Pharmaceuticals (Basel). 2023 Apr 15;16(4):596. doi: 10.3390/ph16040596 (PMC10143156; doi:10.3390/ph16040596)
Supplement: Supplementary file 1 [file pharmaceuticals-16-00596-s001.zip › Table S1 Analysis Results.pptx]

## Slide 1
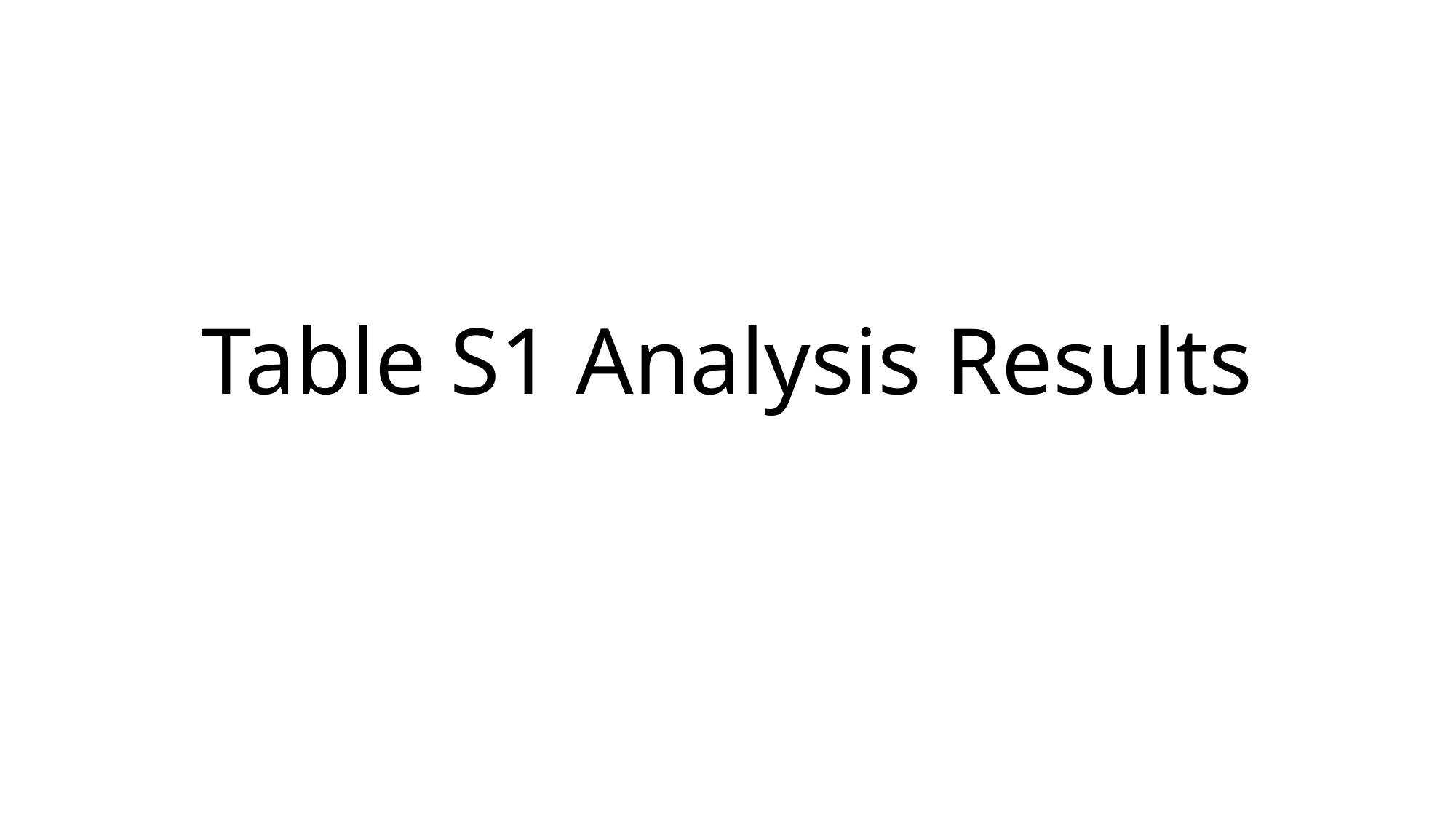

# Table S1 Analysis Results

## Slide 2
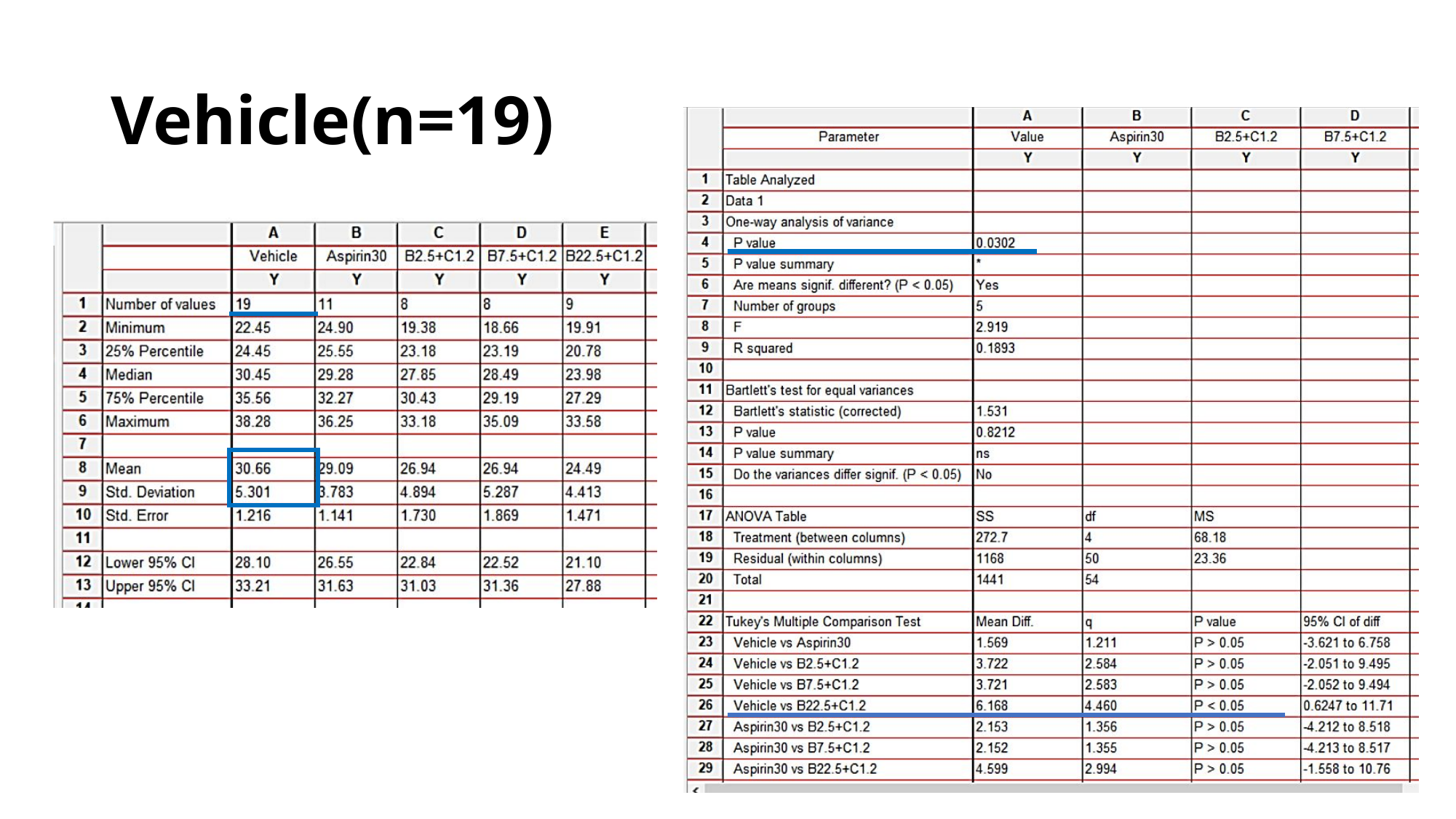

# Vehicle(n=19)

## Slide 3
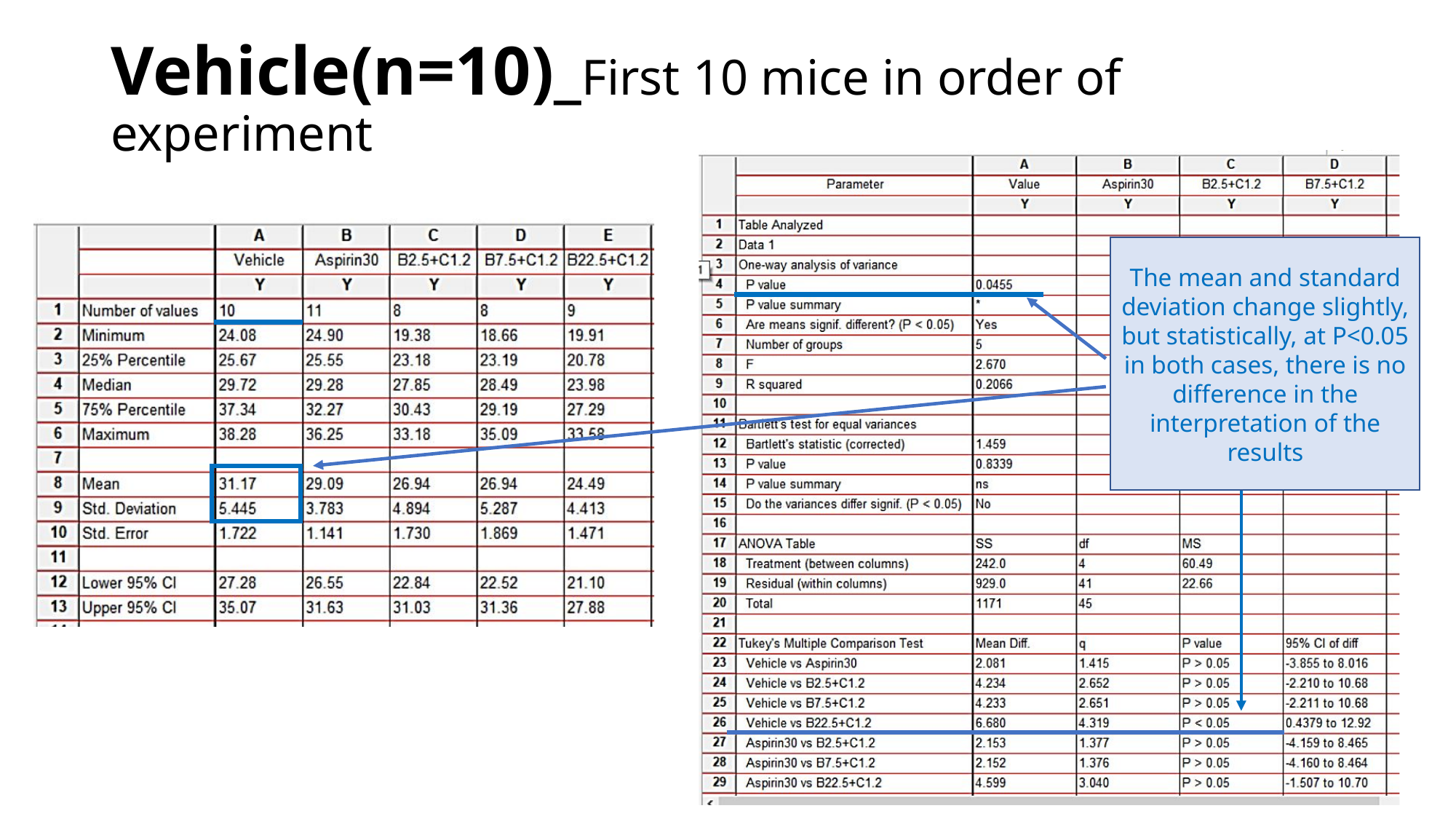

# Vehicle(n=10)_First 10 mice in order of experiment
The mean and standard deviation change slightly, but statistically, at P<0.05 in both cases, there is no difference in the interpretation of the results

## Slide 4
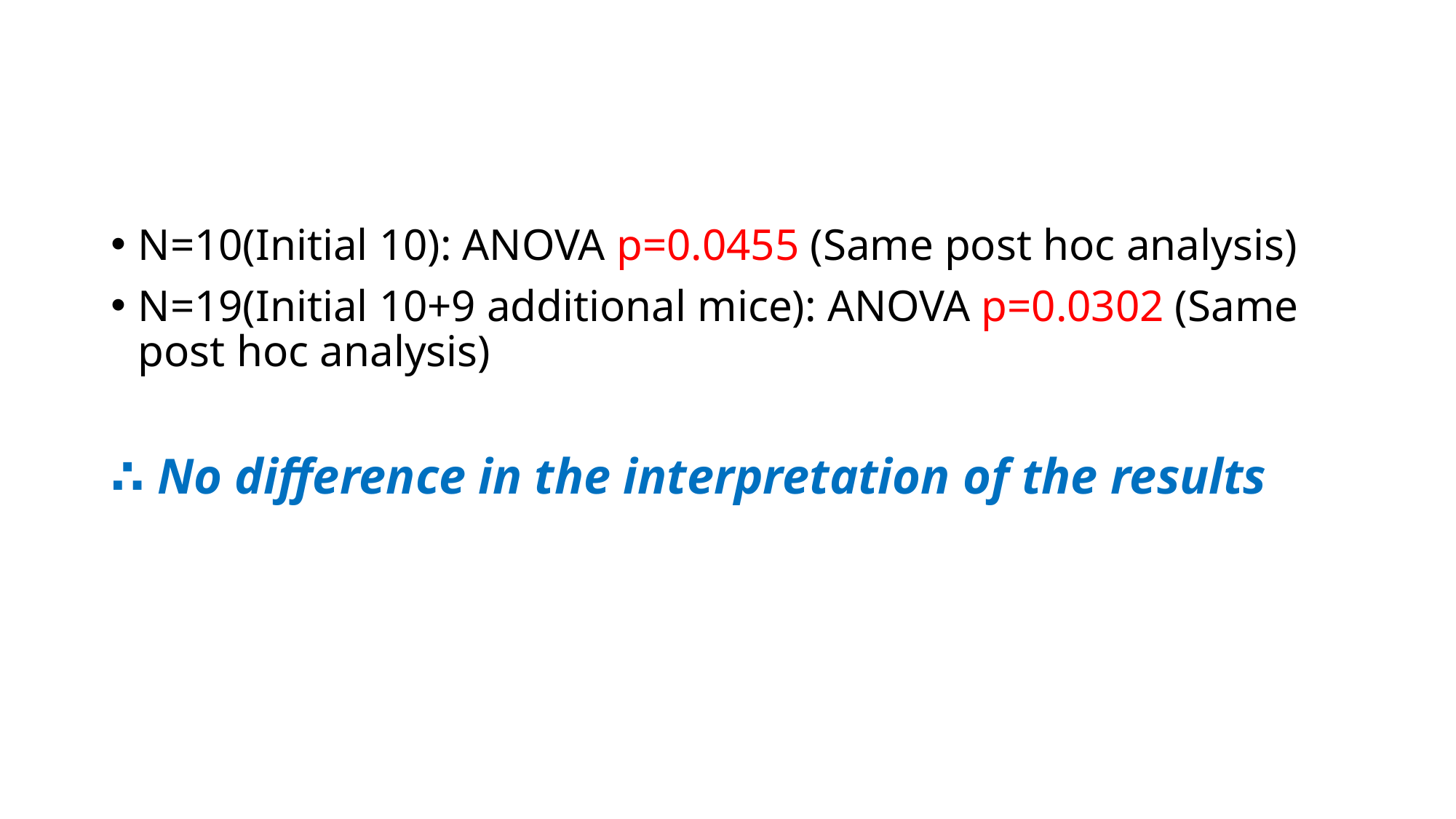

#
N=10(Initial 10): ANOVA p=0.0455 (Same post hoc analysis)
N=19(Initial 10+9 additional mice): ANOVA p=0.0302 (Same post hoc analysis)
∴ No difference in the interpretation of the results
